# Supplementary figures and images for: Genome-Wide Identification and Characterization of TCP Gene Family Members in Melastoma candidum
Source: Molecules. 2022 Dec 18;27(24):9036. doi: 10.3390/molecules27249036 (PMC9787641; doi:10.3390/molecules27249036)

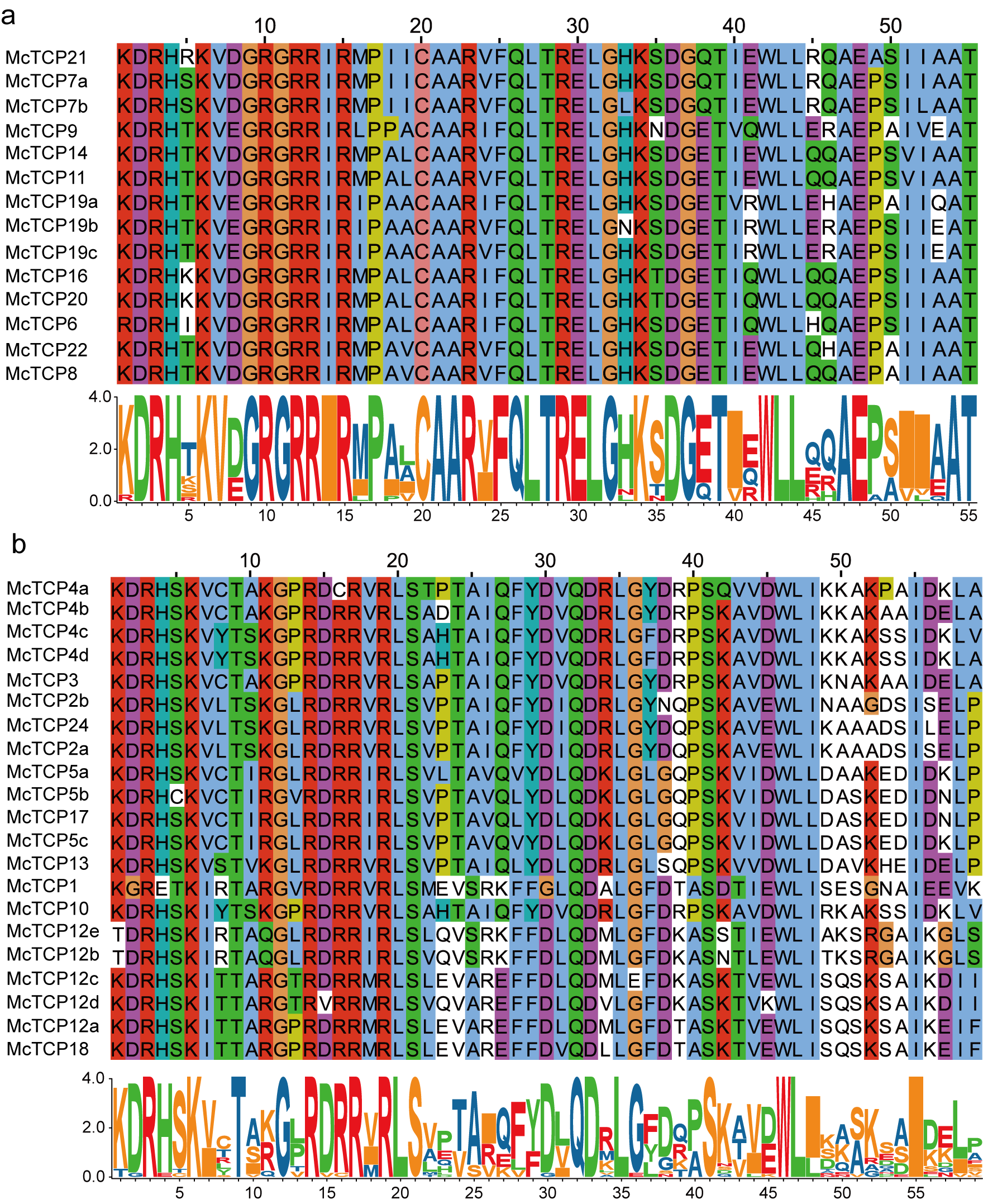

Supplement: Supplementary file 1 [file molecules-27-09036-s001.zip › Figure S1.tif]
